# French Translation and Validation of the Rating-of-Fatigue Scale

**DOI:** 10.1186/s40798-021-00316-8

**Published:** 2021-04-08

**Authors:** Callum G. Brownstein, Diana Rimaud, Benjamin Singh, Laurie-Anne Fruleux-Santos, Marine Sorg, Dominic Micklewright, Guillaume Y. Millet

**Affiliations:** 1 Univ Lyon, UJM-Saint-Etienne, Inter-university Laboratory of Human Movement Biology, EA 7424, F-42023 Saint-Etienne, France; 2 Department of Exercise and clinical Physiology, University Hospital of Saint-Etienne, Saint Etienne, France; 3 School of Sport, Rehabilitation and Exercise Sciences, University of Essex, Wivenhoe Park, Colchester, Essex, CO4 3SQ UK; 4 Institut Universitaire de France (IUF), Paris, France; 5 Laboratoire Interuniversitaire de Biologie de la Motricité, Bâtiment IRMIS, 10 rue de la Marandière, 42270 Saint Priest en Jarez, France

## Abstract

**Background:**

The Rating of Fatigue (ROF) scale can measure changes in perceived fatigue in a variety of contexts.

**Objective:**

The aim of the present study was to translate and subsequently validate the ROF scale in the French language.

**Methods:**

The study was composed of three phases. Phase 1 involved a comprehensive translation, back-translation, and consolidation process in order to produce the French ROF scale. During phase 2, the face validity of the French ROF scale was assessed. A cohort of 60 native French speaking participants responded to a range of Likert scale items which probed the purposes of the ROF scale and what it is intended to measure. During phase 3, the convergent and divergent validity of the ROF scale was assessed during ramped cycling to exhaustion and 10 min of resting recovery.

**Results:**

The results from phase 1 demonstrated comparability and interpretability between the original and back-translated ROF scale. In phase 2, participants reported a high face validity, with a score of 3.48 ± 0.70 out of 4 when given the item probing whether the scale “measures fatigue”. This score further improved (3.67 ± 0.57, *P* = 0.01) after participants read the accompanying instructions. Participants were able to distinguish the purposes of the scale for measuring fatigue rather than exertion. In phase 3, strong correlations were found between ROF and heart rate (HR) both during exercise (*r* = 0.91, *P* < 0.01) and recovery (*r* = 0.92, *P* < 0.01), while discriminant validity between ROF and rating of perceived exertion (RPE) was found during recovery.

**Conclusion:**

The present study permits the applications of the ROF scale in the French language.

**Supplementary Information:**

The online version contains supplementary material available at 10.1186/s40798-021-00316-8.

## Keypoints

- The Rating of Fatigue (ROF) scale has demonstrated high levels of face and construct validity and is thus a valid and practical tool to assess fatigue in a variety of contexts.
- The ROF scale has never before been validated in the French language.
- The present study performed a thorough translation and cross-cultural adaptation procedure of the ROF scale and demonstrated that the translated scale maintains high levels of face and construct validity.

## Introduction

The study of fatigue has captivated researchers from a wide range of disciplines for centuries. Likely due in part to the diversity in the fields of research across which fatigue is studied, defining fatigue has proved a problematic and contentious issue. Indeed, numerous definitions exist both across and within disciplines, ranging from those in the exercise-sciences defining fatigue as a reduction in maximum force generating capacity of the muscle [1], to those in psychological fields defining fatigue as an overwhelming sense of tiredness, lack of energy and a feeling of exhaustion [2]. The wide-ranging definitions and applications of the term fatigue have been criticised for nebulising our theoretical understanding of fatigue [3] and limiting the translation of this knowledge towards improved human performance [4]. Accordingly, recent efforts have been made to provide a universal definition of fatigue, applicable to both athletic and clinical populations, which encompasses the interdependent physical and cognitive processes that occur with numerous chronic health conditions, and during and following strenuous exercise [4]. To this end, Enoka and Duchateau [4] defined fatigue as a debilitating symptom of tiredness and weakness, dictated by interactions between performance fatigability, which involves an acute exercise-induced reduction in force and power output of the involved muscles, and perceived fatigability, involving changes in sensations that accompany fatigue.

Much like the numerous definitions of fatigue which exist in the literature, various instruments have been used to measure fatigue across a range of populations, such as the Total Recovery Quality scale [5] and Recovery-Stress Questionnaire for athletes [6], Functional Assessment of Cancer Therapy for cancer patients [7] and The Fatigue Descriptive Scale for multiple-sclerosis patients [8], with these scales often designed to capture population-specific symptoms. While using population-specific questionnaires to assess fatigue has its merits, the heterogeneity in the measurement tools impedes inter-pathological comparisons and the development of a generalised theory of fatigue [3]. Recently, Micklewright et al. [3] developed and validated a general “Rating-of-Fatigue” (ROF) scale, an 11-point scale with accompanying descriptors which is capable of tracking fatigue across any range of daily life, physical activity and recovery contexts. Specifically, the ROF scale demonstrated high levels of face and construct validity during ramped exercise and resting recovery, and to assess circadian and circaseptan (weekly) variations in fatigue [3]. Moreover, the ROF demonstrated discriminant validity from the Rating of Perceived Exertion Scale (RPE) [9] during post-exercise recovery, whilst also correlating well with physiological markers during the recovery period [3]. This is an important finding considering that previous work has used the RPE to track recovery post-exercise [10, 11], and highlights the divergence between the constructs of exertion and fatigue, thereby emphasising that RPE should not be used in an attempt to capture momentary fatigue. Overall, the ROF scale offers advantages due to its practicality regarding the speed and ease of use, and its ability to capture momentary fatigue, rather than relying on participants to recall their level of fatigue over previously defined periods. Thus, the ROF offers a promising instrument to measure fatigue, and several studies have implemented this tool in subsequent research [12–14].

Given the wide-ranging potential applications of the ROF scale, there is a requirement to translate and demonstrate the cross-cultural validity of the scale in other languages. Simply translating a scale from one language to another without consideration for potential cross-cultural and ethnic differences is deemed inappropriate [15]. Specifically, it is recommended that thorough translation, back-translation and consolidation procedures are performed when translating scales and questionnaires [15]. Moreover, testing for face and construct validity is recommended to ensure that the translated scale or questionnaire maintains validity and measurement properties required for the intended application [15, 16].

French is the fifth most widely spoken language in the world, spoken by more than 274 million people in countries such as France, Belgium, Canada, Switzerland and Africa. At present, no study exists validating the ROF scale in French. Accordingly, the present study aimed to validate the ROF scale in French to permit its use during future research in the French language.

## Methods

### Design

**Fig. 1:**
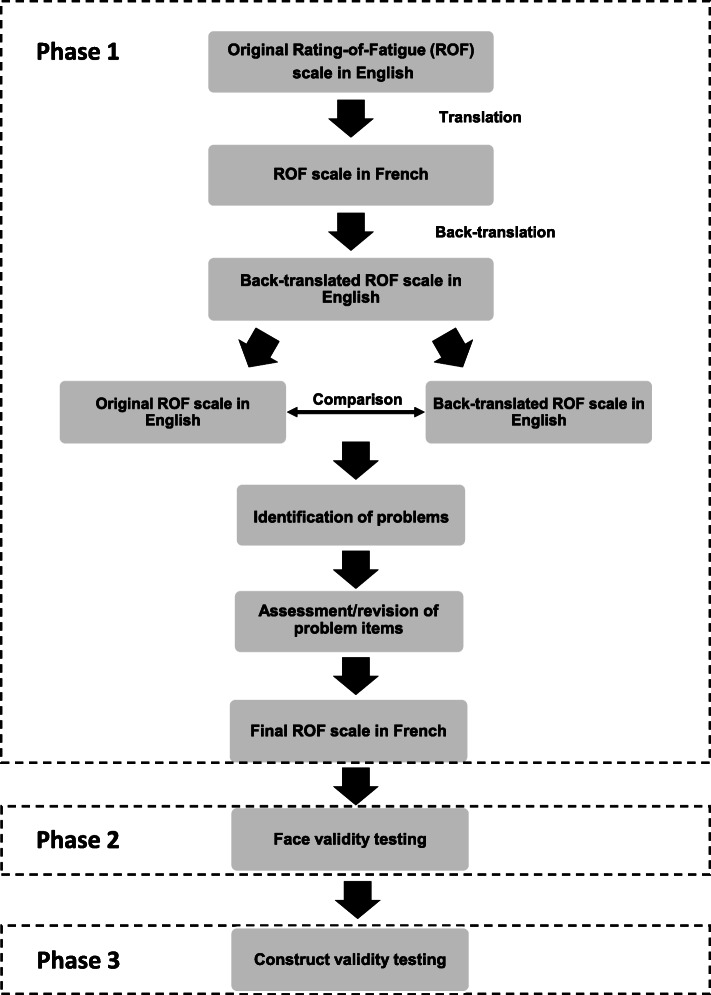
Flowchart of the study design, including the translation (phase 1), validation (phase 2) and construct validity (phase 3) processes of the Rating of Fatigue scale in the French language

The study received institutional ethical approval from the University Jean Monnet, Saint Etienne Ethics committee in accordance with the ethical standards established in the Declaration of Helsinki (submission reference: IRBN682020/CHUSTE). All participants involved in the study provided informed consent to participate. In order to translate and validate the ROF scale in French, the study was divided into three phases: (1) translation, back-translation and consolidation of ROF scale, (2) face validity testing following translation and, (3) construct validity testing. A flow chart of the study procedures is displayed in Fig. 1.

### Phase 1—Translation, Back-translation and Consolidation

The translation phase involved three stages based on the recommended approach for cross-cultural adaptation of study instruments [15, 17], including initial translation to French, followed by back-translation of the French version to English and a formal comparison of the original version of the ROF scale with the back-translated version. For the initial phase, two bilingual native French speakers who were also fluent in the English language were asked to independently translate both the ROF scale and the accompanying instructions provided with the scale [3] from the English to French language. The two translations were then assessed by two native French speakers, who reached a consensus on any discrepancies to produce a single translated scale in the French language. Next, three bilingual native English speakers who were also fluent in the French language independently back translated the French version of the scale to English. The native French and English speakers were independent of the authors, were unaware of the purposes of the study and were not familiar with the ROF scale. Based on the method for validating translated instruments developed by Sperber et al. [18], the original ROF scale in the English language was compared with the three back-translated, source language versions, to be examined for any discrepancies. This procedure involved using the “Comparability/Interpretability Rating Sheet” [18] (Table 1). Using this instrument, every item in the three back-translated English versions of the scale was ranked for comparability of language and similarity of interpretability with the original source language ROF scale. The comparability of language denotes the formal similarity of the words, phrases and sentences, while the interpretability involves the assessment of the degree to which the two versions incorporate the same response, even if the wording differs [18, 19]. For both comparability and interpretability, a Likert scale ranging from 1 (extremely comparable/extremely similar) to 7 (not at all comparable/not at all similar) was used. A panel of four investigators, including two native English speakers, conducted the formal translation review using the Comparability/Interpretability Rating Sheet. If an average score of greater than 3 was obtained for comparability and interpretability, corrections were made to the ROF translated version to ensure comprehension and cultural sensitivity [18]. Once adjustments had been made, the final translated French version of the ROF scale and accompanying instructions was finalised (Additional file 1: Appendix A).

**Table 1:** Comparability/interpretability rating sheet. From Sperber et al. [18]

		*(A) Comparability of language*						
		Extremely comparable	Moderately comparable				Not at all comparable	
English ROF version (Mickelwright et al. 2017)	Back-translated English ROF scale	1	2	3	4	5	6	7
		*(B) Similarity of interpretation*						
		1	2	3	4	5	6	7

### Phase 2—Face Validity Testing with French Rating-of-Fatigue Scale

Using the translated French version of the ROF scale, face validity testing was then performed using methods adapted from that of Micklewright et al. [3], where were consistent with guidelines on face validity testing [20]. Sixty native French speaking participants (26 females) were recruited for this phase of the study (age 47 ± 11 years). Participants included sport and exercise science academics (*n* = 14), non-academic sports or physical activity professionals (*n*=13), medical or health care professional (*n*=11), academics from non-sport disciplines (*n* = 6) and others (*n*=16). All of the participants were selected because of their varying levels of expertise in sport and exercise science, and because of their familiarity with participating in physical activity, exercise and sport. Face validity is a subjective assessment of whether an instrument measured what it is purported to, which for the ROF scale is the perceived level of fatigue. For this phase, 60 native French speaking participants were asked to rate what they thought the ROF scale measured by responding to five questionnaire items, rating them using a 5-point Likert scale (Strongly agree, Agree, Undecided, Disagree and Strongly Disagree from 4 to 0, respectively). The questions probed the extent to which the ROF: (i) represents fatigue, (ii) represents exertion, (iii) descriptive components make the scale easy to understand (iv) descriptive components assist in deciding upon a rating and (v) overall scale is difficult to understand. These questions were derived from the original ROF scale validation by Micklewright et al. [3], with the three questions concerning the diagrammatic components and visual appearance from their study omitted since these are not relevant to translation and subsequent interpretation. Participants were then given the instruction sheet to read, before re-inspecting the ROF scale again. They then completed the same five Likert scales described previously, plus an additional item concerning the usefulness of the instructions in understanding the scale.

### Phase 3—Convergent and Discriminant Validity Testing with French Rating-of-Fatigue Scale During Ramped Cycling to Exhaustion and Resting Recovery

Following face validity testing, the construct validity of the French Rating-of-Fatigue scale was tested. For this phase, 19 participants (6 females, age 46 ± 15, stature 170 ± 10 cm, mass 69 ± 10 kg) were recruited during regular consultations to the Sports Medicine Department at St-Etienne University Hospital. Inclusion criteria for phase 3 included age (18–70 years) and being free from any neurological, rheumatological, cardiovascular, respiratory or metabolic disease, from any traumatic lesions, or from any functional impairment affecting cycling. Participants completed a ramp incremental cycling exercise test to exhaustion on a cycle ergometer (Monark, Ergomedic 839E, Varberg, Sweden) and subsequently remained seated on the cycle ergometer for 10 min while recovery measurements were taken. Participants self-reported physical activity levels were recorded and ranged from sedentary to highly active. The starting power output during cycling was 60 W for women and 80 W for men, with a step increment of 20 W/2 min for women and 30 W/2 min for men. Throughout the cycling exercise, heart rate (HR; Cosmed Quark T12x ECG monitor, Rome, Italy), power output, and a ROF and RPE score were recorded every 100 s. During recovery, HR and a ROF and RPE were recorded every 120 s up to 10 min. The RPE score was derived from the Borg 6–20 scale [21]. Two objective testing methods were used during both ramped exhaustive cycling exercise and 10 min of recovery: (i) convergent validity in which associates were made between ROF measurements and HR, power output and RPE; (ii) discriminant validity by measuring the degree to which ROF and RPE diverge.

### Statistical Analysis

Statistical analyses were performed using Jamovi statistical software (the jamovi project, (2019). *Jamovi* (version 1.0) [Computer Software]. Retrieved from https://www.jamovi.org). For face validity testing, all Likert scale questionnaire responses were scored from 0 to 4, with 0 representing low face validity and 4 representing high face validity for question i (scale measures fatigue). The scores in response to the five questions given before and after the administration of the instruction sheet were compared using non-parametric Wilcoxon’s signed rank test. For convergent validity testing, all variables were expressed in relation to the percentage of time to exhaustion, whereby 0% represents the beginning of the ramped cycling test and 100% represents the point of volitional exhaustion. In order to provide a continuous scale during recovery, recovery time was also expressed as a percentage of time to exhaustion whereby the point of fatigue occurred at 100% and recovery time as a percentage increase in time relative to time to exhaustion [3]. For each participant, a Pearson’s product moment correlation was calculated for each ROF measure against HR, power output and RPE during exercise, and for HR and RPE during recovery. The individual *r* values were subjected to a single-sample *t* test across the participant group. All outcomes are presented as mean ± SD, and statistical significance was set at an *α* level of < 0.05. Cohen’s *d* effect sizes are also provided.

## Results

### Phase 1—Translation, Back-translation and Consolidation

A French version of the ROF scale was obtained through two translators. The translation version was back-translated to English by three other translators, and comparisons made between the original version and the back-translated versions. Only two sentences from the instruction sheet of one of the three back-translated versions obtained an average comparability/interpretability score > 3. Specifically, the primary issue arising from the back-translated version of the scale was that two of the three back-translations used the words “tired” instead of “fatigued”. While these words have distinct definitions in the English language, the word for “tired” and “fatigued” in the French language is the same (fatigué), and can be used either in the context of physical exertion, or to describe tiredness and weariness. The average scores for comparability and interpretability of the back-translated and original ROF scale instruction sheet were 2.2 and 1.7, respectively. For the ROF scale itself, the average scores for comparability/interpretability were 1.7 and 1.6, respectively. Thus, modifications to the translated French ROF scale were minor.

### Phase 2—Face Validity Testing with French Rating-of-Fatigue Scale

**Fig. 2:**
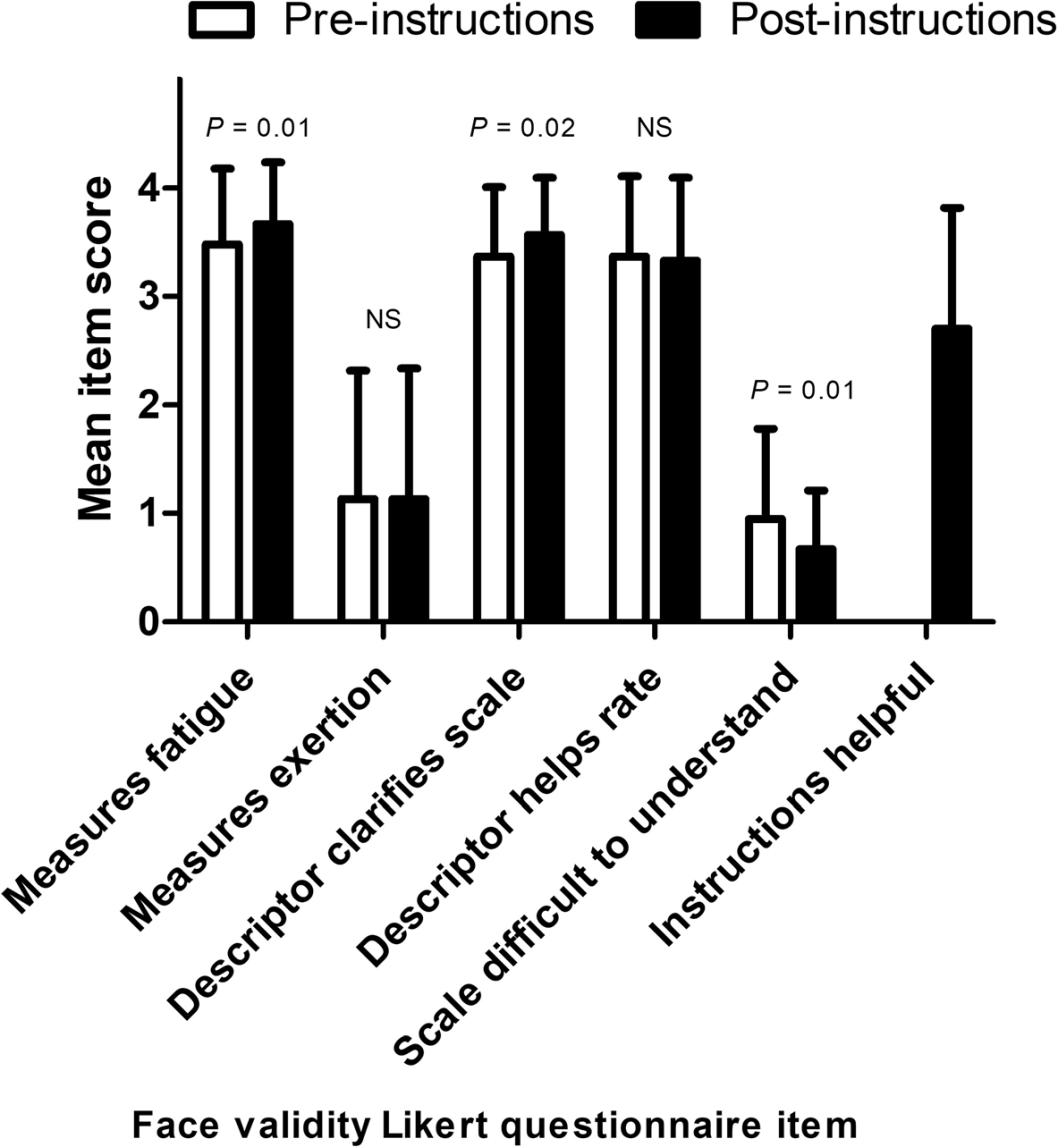
Face validity outcomes of the rating-of-fatigue scale before and after the scale instructions

Scores for the face validity Likert questionnaires are displayed in Fig. 2. A high level of face validity was found, as demonstrated by the high mean Likert score for question i (scale measures fatigue). The score for question i increased further following the reading of instructions (pre: 3.48 ± 0.70 vs. post: 3.67 ± 0.57; *P* = 0.01; *d* = 0.47), indicating that the instructions assisted in clarifying the purposes of the scale. Low scores were demonstrated for question ii (scale measures exertion), which did not change after reading instructions (pre: 1.13 ± 1.19 vs. post: 1.13 ± 1.21; *P* = 0.92, *d* = 0.00). Descriptors were perceived as helpful to clarify the scale, with a further improvements improvement following the reading of instructions (pre: 3.37 ± 0.64 vs. post: 3.57 ± 0.53; *P* = 0.02, *d* = 0.32). These descriptors were also perceived as helpful when deciding how to rate the scale, with no improvement following instruction (pre: 3.37 ± 0.75 vs. post: 3.33 ± 0.77; *P* = 0.82, *d* = 0.04). Low scores were demonstrated in response to the item “scale is difficult to understand”, with this score further reduced after reading instructions (pre: 0.95 ± 0.83 vs. post: 0.67 ± 0.54; *P* = 0.01, *d* = 0.35). A score of 2.70 ± 1.12 was obtained for the item “instructions are helpful”.

### Phase 3—Convergent and Discriminant Validity Testing with French Rating-of-Fatigue Scale During Ramped Cycling to Exhaustion and Resting Recovery

**Fig. 3:**
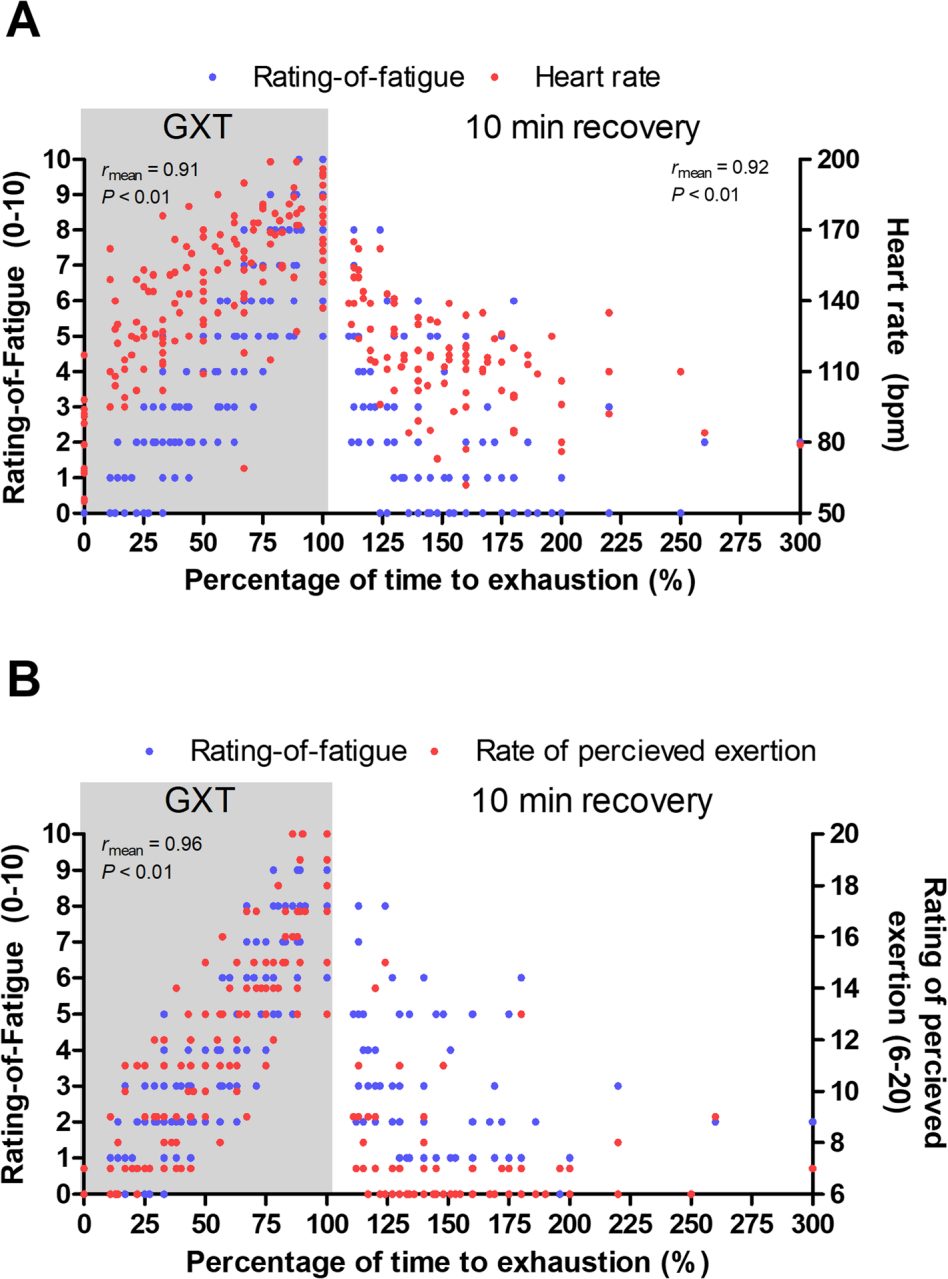
Relationship between ratings of fatigue and heart rate **a** and perceived exertion **b** during graded cycling exercise (GXT) and 10 min of resting recovery

During the cycling test, strong correlations were found between the ROF scale and HR, RPE and power output (Table 2). Similarly, strong correlations were found between the ROF scale and HR during the recovery period (Table 2). However, during recovery, the ROF scale exhibited divergent validity against RPE, and correlation calculations were not possible since almost all recorded RPE scores were 6 without any variance. The associations between ROF and HR and RPE during graded cycling and recovery are presented in Fig. 3a and b, respectively.

**Table 2:** Mean Pearson product moment correlation coefficients between Rating-of-Fatigue scale and HR, RPE and power output

	Mean Pearson coefficients		Single sample ***t*** test outcomes		
	*r* mean	SD of *r* mean	*t*(18)	*P*	*d*
*Graded exercise*					
Heart rate	0.91	0.05	73	< 0.01	16.8
Power output	0.97	0.06	68	< 0.01	15.7
RPE	0.96	0.02	18	< 0.01	38.9
*Recovery*					
Heart rate	0.92	0.07	55	< 0.01	12.5

## Discussion

The aim of the present study was to validate the ROF scale (Micklewright et al. 2017) in the French language using recommended guidelines for cross-cultural adaptation of study instruments [15, 18]. In phase 1 of the study, following translation and back-translations, the scores obtained from the comparability and interpretability scoring sheet (Table 1) [18] were 2.2 and 1.7, respectively, for the questionnaire instructions, and 1.7 and 1.6, respectively, for the ROF scale. Thus, comparability and interpretability were demonstrated for both the ROF scale and the accompanying instruction sheet. The primary issue arising from the back-translated version of the scale was that two of the three back-translations used the words “tired” instead of “fatigued”. While these concepts are related, they have distinct definitions in the English language, with tiredness defined as “the state of wishing for sleep or rest” (Oxford English Dictionary, 2011), while fatigue, as defined above, is a debilitating symptom of tiredness and weakness, dictated by interactions between performance fatigability, which involves an acute exercise-induced reduction in force and power output of the involved muscles, and perceived fatigability, involving changes in sensations that accompany fatigue [4]. Thus, the word “tired” is less appropriate to describe the sensations incurred by strenuous exercise. However, in the French language, the word for “tired” and “fatigued” is the same (fatigué), and can be used either in the context of physical exertion, or to describe tiredness and weariness. Consequently, the word “fatigué” was deemed appropriate for the translated ROF scale.

Subsequently, in phase 2 of the study, the face validity of the newly translated French ROF scale was assessed using Likert questionnaires, using similar methods to the original study validating the ROF scale [3] based on guidelines for face validity testing [20]. A high level of face-validity was demonstrated, as indicating by the high score on the item probing whether the scale measures fatigue. The accompanying instructions further improved clarity on the purposes of the scale, and these instructions should thus be provided when using the scale to facilitate comprehension. The low score obtained for the item probing whether the scale represents exertions indicate that participants are able to distinguish the constructs of fatigue from exertion, as demonstrated by Micklewright et al. [3]. Specifically, whilst exertion and fatigue are highly correlated during physical exertion, they are distinct during recovery following exercise, in that fatigue remains elevated whilst perceived exertion is diminished [3]. Furthermore, fatigue which is accumulated throughout the day is perceived at rest, in the absence of exertion [3], and it is thus important that participants are able to distinguish these two concepts. Finally, the translated descriptors were reported as helpful to clarify the scale and to choose and appropriate response.

In phase 3 of the present study, the construct validity of the French translated ROF scale was assessed during an incremental ramp cycling exercise protocol. Corroborating the findings of Micklewright et al. [3], ROF during exercise and recovery was strongly correlated with the physiological measured through HR at various stages of exercise and recovery and was similarly correlated with the external demands measured through power output during cycling. Furthermore, the divergence between ROF and RPE during the recovery period in phase 3 highlights that the ROF scale maintains discriminant validity from perceived exertion, as previously demonstrated in the original version of the scale [3]. Accordingly, the results from phases 2 and 3 demonstrate that the French translated ROF both subjectively and objectively maintains its measurement properties and is suitable for application during and following exercise.

## Conclusion

The present study translated the Rating of Fatigue scale to the French language, and, using recommended cross-cultural adaptation methods, demonstrated extreme comparability and interpretability with the original Rating of Fatigue scale in the English language. Subsequently, using recommended face validity testing methods, the study demonstrated that the translated scale has a high level of face validity when measuring fatigue. Finally, the convergent validity between the Rating of Fatigue scale and physiological and external demands of cycling was demonstrated both during exercise and recovery, while the divergent validity between Rating of Fatigue and ratings of perceived exertion was maintained using the translated version of the scale. Thus, the scale maintains subjective and objective measurement properties demonstrated by the original Rating of Fatigue scale. Future studies can implement the newly translated scale and should do so by having participants carefully visually inspect the scale and reading the instructions before providing a response. Given the widespread potential applications of the Rating of Fatigue scale in all populations, and the requirement to perform appropriate cross-cultural adaptation when translating study instruments, the present study is of importance and permits the application of the Rating of Fatigue scale amongst French speaking participants in future research.

## Supplementary Information

**Additional file1: APPENDIX A.** – The French Rating-of-Fatigue Scale and Instructions

## Data Availability

The datasets generated during and/or analysed during the current study are available from the corresponding author on reasonable request.

## Abbreviations

HR: Heart rate
ROF: Rating of fatigue
RPE: Rating of perceived exertion
SD: Standard deviation
