# Supplementary material for: French Translation and Validation of the Rating-of-Fatigue Scale
Source: Sports Med Open. 2021 Apr 8;7:25. doi: 10.1186/s40798-021-00316-8 (PMC8026791; doi:10.1186/s40798-021-00316-8)
Supplement: Supplementary file 1 — Additional file1: APPENDIX A. – The French Rating-of-Fatigue Scale and Instructions [file 40798_2021_316_MOESM1_ESM.docx]

**APPENDIX A – The French Rating-of-Fatigue Scale and Instructions**

**Echelle d’évaluation de la fatigue**

**Echelle d’évaluation de la fatigue et instructions**

L’échelle d’évaluation de la fatigue (EDF) vous permettra d’évaluer à quel point vous vous sentez fatigué. L’échelle peut vous être présentée par une autre personne ou, dans certaines circonstances, il peut vous être demandé de la remplir vous-même. Quelle que soit la méthode utilisée, il est important que vous lisiez dans un premier temps les instructions suivantes :

1. Veuillez maintenant vous familiariser avec l’EDF en la regardant attentivement. Vous remarquerez qu’elle comporte 11 points numériques compris entre 0 et 10. Il y a également 5 légendes et 5 schémas qui sont destinés à vous aider à comprendre l’échelle et à faire votre évaluation.
2. Lorsque l’on vous présente l’EDF, veuillez l’observer avec attention avant de donner votre réponse numérique comprise entre 0 et 10. Essayez toujours de répondre le plus honnêtement possible, en donnant une évaluation qui reflète au mieux votre perception de la fatigue à ce moment précis.
3. Essayer de ne pas trop hésiter et faites en sorte de ne donner qu’UN seul chiffre comme réponse. Par exemple, éviter de répondre en donnant deux chiffres comme ‘trois ou quatre’.
4. Veuillez maintenant lire les exemples d’évaluation de l’EDF suivants :

- Une réponse “0” indiquerait que vous ne vous sentez pas du tout fatigué. Par exemple, cela pourrait être peu de temps après votre réveil suite à une bonne nuit de sommeil. Maintenant, essayez de penser à une occasion similaire dans le passé où vous avez éprouvé le plus bas niveau de fatigue et utilisez-la comme référence.
- Une réponse “10” indiquerait que vous vous sentez complètement fatigué et épuisé. Par exemple, cela pourrait être une incapacité à rester éveillé, comme tard dans la nuit, mais cela pourrait aussi correspondre à une situation de sprint où vous n’auriez plus les ressources physiques pour continuer. A nouveau, essayez de penser à un exemple similaire que vous avez réellement vécu dans le passé.

**
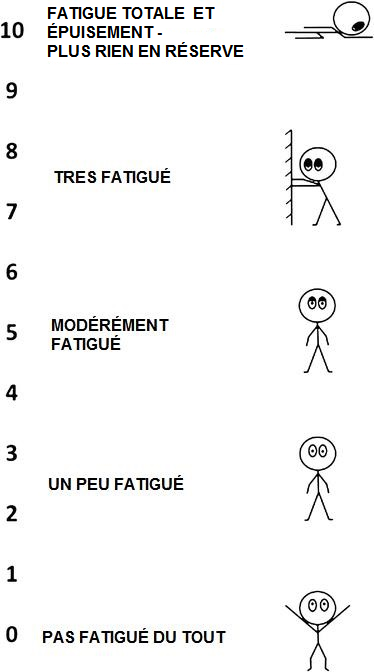
**
